# Promising effects of high-intensity focused ultrasound combined with eribulin and sintilimab in a patient with advanced leiomyosarcoma: a case report and literature review

**DOI:** 10.3389/fonc.2026.1775289

**Published:** 2026-07-09

**Authors:** Shaoli Li, Menglei Jin, Qunan Sun, Hui Wang, Xiaoye Hu, Rui Bai, Ying Dong

**Affiliations:** 1 Department of Medical Oncology, the Second Affiliated Hospital, Zhejiang University School of Medicine, Hangzhou, China; 2 Key Laboratory of Cancer Prevention and Intervention, Ministry of Education, the Second Affiliated Hospital, Cancer Institute, Zhejiang University School of Medicine, Hangzhou, China; 3 Cancer Center, Zhejiang University, Hangzhou, China; 4 Department of Clinical Oncology, Li Ka Shing (LKS) Faculty of Medicine, the University of Hong Kong, Hong Kong, China; 5 Division of Hepatobiliary and Pancreatic Surgery, Hepatobiliary and Pancreatic Interventional Treatment Centre, The First Affiliated Hospital, Zhejiang University School of Medicine, Hangzhou, China; 6 Department of Pathology, the Cancer Hospital of the University of Chinese Academy of Sciences (Zhejiang Cancer Hospital), Institute of Basic Medicine and Cancer (IBMC), Chinese Academy of Sciences, Hangzhou, China

**Keywords:** advanced leiomyosarcoma, combination therapy, eribulin, high-intensity focused ultrasound, sintilimab

## Abstract

Leiomyosarcoma (LMS) is an aggressive malignancy characterized by a high risk of recurrence and metastasis. The prognosis for advanced LMS remains poor due to limited effective treatment options. We report a case of advanced bladder LMS in a male patient whose disease progressed following standard therapies including surgery, first-line chemotherapy, and anlotinib. Disease stabilization was achieved after two cycles of eribulin combined with sintilimab. Subsequently, a partial response was achieved after the addition of high-intensity focused ultrasound (HIFU) to this regimen, and the response was maintained for 7.2 months with continued eribulin and sintilimab. This sustained disease control suggests that the integration of HIFU may augment the antitumor activity of systemic therapy in advanced LMS, warranting further investigation into this multimodal approach for patients refractory to conventional treatments.

## 1 Introduction

Leiomyosarcoma (LMS) is one of the most common subtypes of soft tissue sarcomas (STS), accounting for approximately 10-20% of newly diagnosed cases (1). For localized disease, wide resection with negative margins remains the cornerstone of treatment, with adjuvant radiotherapy or chemotherapy considered in high-risk cases. Nevertheless, LMS often follows an aggressive clinical course, with a high propensity for local recurrence and distant metastasis (2). The management of advanced LMS is particularly challenging due to limited systemic options and generally poor outcomes, highlighting the urgent need for novel therapeutic strategies.

Here, we present the case of a 49-year-old man with advanced LMS of the bladder who developed metastatic disease after surgery and first-line chemotherapy. Following progression on anlotinib, the patient achieved disease stabilization with eribulin and sintilimab. Notably, the subsequent addition of HIFU was associated with a marked tumor reduction, suggesting a potential synergistic effect of combined local and systemic therapy.

## 2 Case presentation

**Figure 1:**
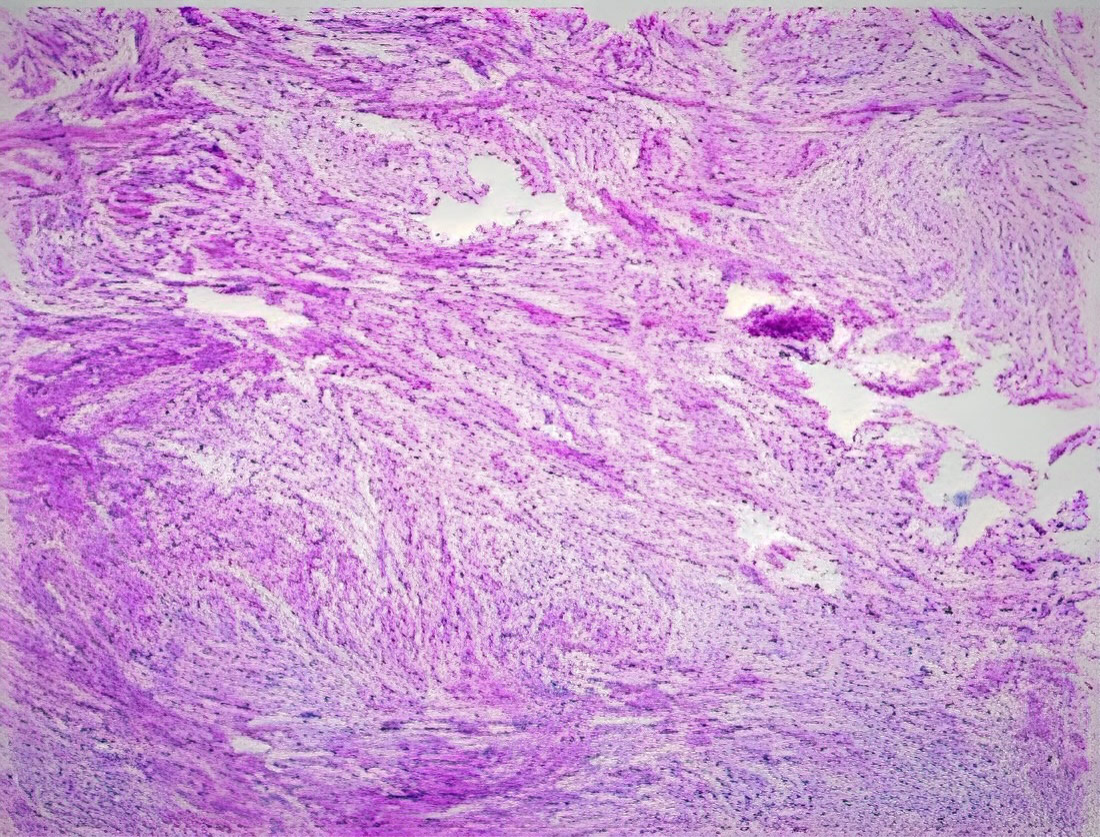
Histopathological findings of the resected bladder leiomyosarcoma (H&E staining). The tumor is composed of intersecting fascicles of spindle-shaped cells with eosinophilic cytoplasm and elongated, blunt-ended nuclei (×200). Mitotic figures are infrequent, and tumor necrosis is absent. Immunohistochemistry (not shown) was positive for SMA and desmin, confirming smooth muscle differentiation.

A 49-year-old male presented with a one-month history of gross hematuria. He had no significant medical or family history. Bladder ultrasound revealed a hyperechoic nodule (1.94 × 1.47 cm) on the anterior wall. Cystoscopic biopsy indicated low-grade LMS. The patient underwent robotic-assisted radical cystectomy. Histopathological examination of the resected specimen revealed a tumor composed of intersecting fascicles of spindle-shaped cells with abundant eosinophilic cytoplasm and elongated, blunt-ended nuclei (**Figure 1**). Mitotic figures were infrequent (< 5 per 10 high-power fields), and no tumor necrosis was observed. According to the original pathology report, immunohistochemical staining demonstrated diffuse positivity for smooth muscle actin (SMA) and desmin, confirming smooth muscle differentiation. These findings confirmed the diagnosis of low-grade leiomyosarcoma of the bladder. Final pathological staging was T2aN0M0. Regular surveillance was initiated postoperatively.

**Figure 2:**
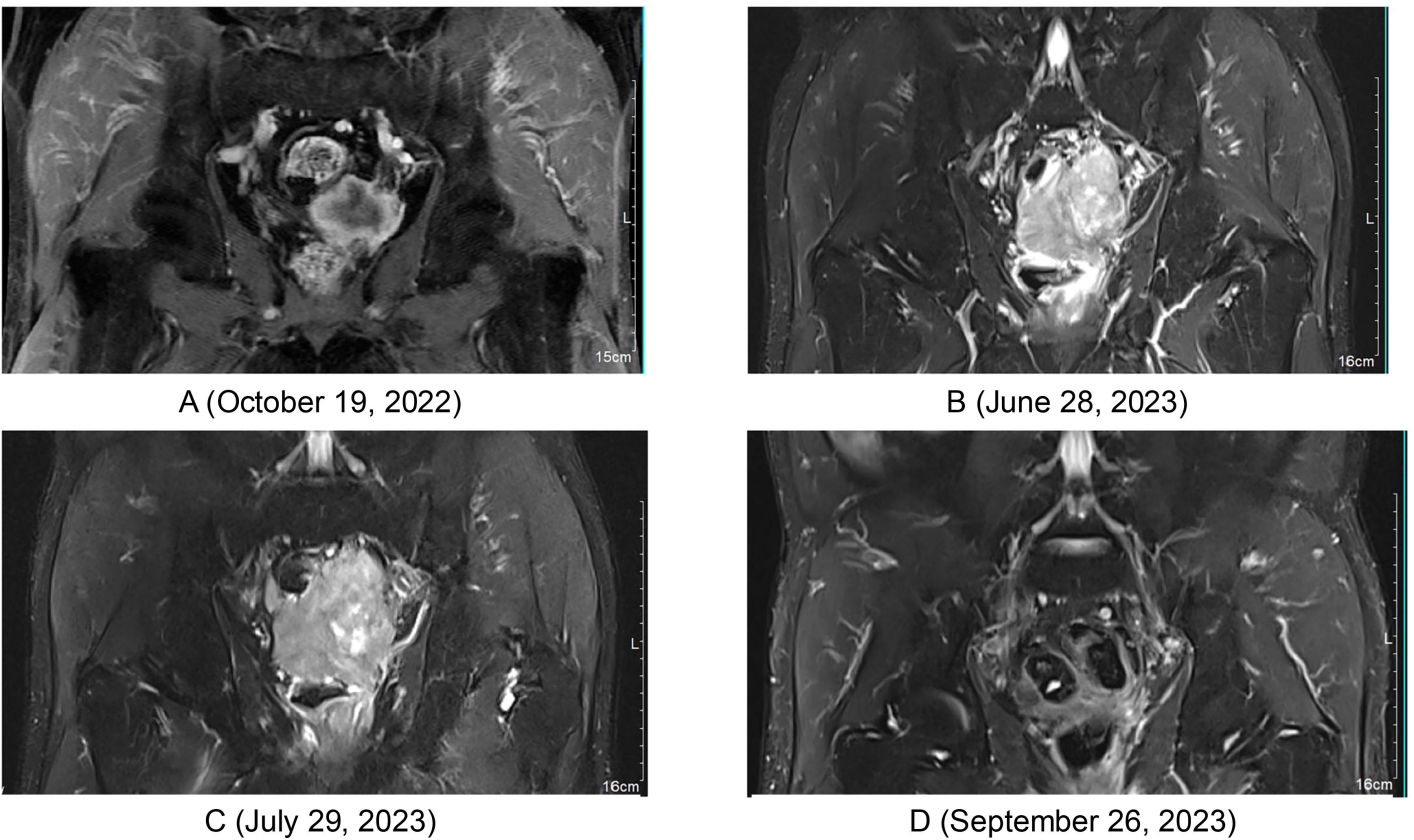
Serial pelvic MRI enhanced scans showing disease course. **(A)** The pelvic MRI enhanced scan showed a mass shadow on the left side of the rectal region, indicating metastasis. **(B)** The pelvic MRI enhanced scan revealed an increase in the size of the mass shadow on the left side of the rectum, indicating disease progression. **(C)** The pelvic MRI enhanced scan indicated stable disease after two cycles of treatment with eribulin and sintilimab. **(D)** The pelvic MRI enhanced scan indicated a significant reduction in tumor size compared to the previous scan after receiving the combination of high-intensity focused ultrasound, combined with eribulin and sintilimab.

Fifteen months after surgery, contrast-enhanced pelvic magnetic resonance imaging (MRI) showed a mass in the left rectal region (**Figure 2A**), suggestive of metastasis. Exploratory laparotomy identified a 2 × 2 cm peritoneal nodule and a larger 4 × 4 cm mass involving the left pelvic and anterior rectal walls, adjacent to the left internal iliac vessels. Due to the high risk of hemorrhage, resection was aborted. Histopathology of the peritoneal nodule confirmed metastatic LMS. The patient received six cycles of epirubicin plus dacarbazine, achieving stable disease. Anlotinib was then initiated but was discontinued after one cycle due to hematochezia; MRI at that time indicated disease progression (**Figure 2B**).

Given the unavailability of trabectedin, the patient was offered gemcitabine plus docetaxel or eribulin plus immunotherapy. He opted for the latter and received two cycles of eribulin plus sintilimab, resulting in stable disease (**Figure 2C**). In light of the persistent tumor burden, HIFU was performed, followed by two additional cycles of eribulin and sintilimab. Treatment was well-tolerated, hematochezia resolved, and follow-up MRI demonstrated a significant tumor reduction (**Figure 2D**), meeting criteria for partial response according to Response Evaluation Criteria in Solid Tumors (RECIST) 1.1.

The patient continued to receive eribulin and sintilimab after HIFU. The partial response was durable, with a progression-free survival of 7.2 months from the initiation of combination therapy. As of April 2026, the patient remains alive, with an overall survival (OS) of 57 months since the initial diagnosis in July 2021.

## 3 Discussion

This case describes a patient with advanced bladder LMS who achieved a durable partial response following treatment with HIFU combined with eribulin and sintilimab, with disease control maintained for over 7 months after prior lines of therapy had failed.

### 3.1 Current treatment landscape for advanced LMS

Up to now, surgical resection with negative margin remains the cornerstone for localized LMS. In the first-line chemotherapy setting, doxorubicin or gemcitabine-based regimens are commonly used (3). A randomized phase III study compared the efficacy of doxorubicin versus gemcitabine plus docetaxel, demonstrating no significant difference in the progression-free-survival (PFS) or overall survival (OS) between the two groups. However, the mean global health status score was numerically higher in the doxorubicin group (4). Interestingly, a previous study showed that patients treated with doxorubicin plus ifosfamide demonstrated longer PFS than doxorubicin alone, although there was no difference in OS (5). Of note, a post-analysis of this trail revealed that the ifofamide provided little benefit for patients with LMS. A multicenter retrospective study explored the activity of doxorubicin alone, doxorubicin plus ifosfamide, and doxorubicin plus dacarbazine as first-line treatment for patients with advanced LMS. The results indicated the combination of doxorubicin and dacarbazine showed favorable activity in terms of both the PFS and OS, which was consistent with the previous studies (6, 7). Additionally, doxorubicin plus trabectedin has demonstrated impressive efficacy as a first-line treatment for patients with advanced LMS (8, 9). A recent phase III study showed that patients with unresectable or metastatic LMS treated with doxorubicin and trabectedin as first-line therapy achieved significantly longer PFS than those treated with doxorubicin alone (10). Other first-line options include gemcitabine alone or in combination with vinorelbine (11).

In the later-line treatment, trabectedin had showed promising efficacy for patients with advanced STS after the failure of doxorubicin treatment (12). Notably, due to the better response in the specific subtypes of STS, trabectedin was approved to be the second-line treatment for patients with advanced LMS and liposarcoma (LPS) (13, 14). Other studies have also explored potential agents for STS patients who have failed first-line treatment. The results suggested that dacarbazine, with or without gemcitabine, could be a choice to consider in the refractory setting for LMS (15). However, a later phase III trial indicated that trabectedin demonstrates superior disease control than dacarbazine in patients with advanced LMS or LPS after prior conventional chemotherapy (16). Another phase III study showed eribulin demonstrated significantly longer OS than dacarbazine for previously treated patients with advanced LPS or LMS. Interestingly, the specific-LMS subgroup analysis revealed comparable efficacy for dacarbazine and eribulin. In addition, benefit with eribulin was stronger in patients with LPS than those with LMS (17), which may account for the reason why eribulin was approved in the second-line treatment for LPS but not for LMS. Of note, the LPS subgroup was small and the comparisons between the subgroups was not statistically powered. In clinical practice, eribulin is still used for later-line treatment of LMS. Apart from chemotherapy, pazopanib was another choice for patients with advanced LMS who have previously received chemotherapy (18, 19). Pazopanib is a small molecular tyrosine kinase inhibitor that has been shown to inhibit KIT, platelet-derived growth factor, and vascular endothelial growth factor (20). A phase III trial included 165 patients with LMS showed that pazopanib had longer PFS than placebo, but there was no significant difference in terms of the OS (18).

In recent years, immunotherapy has made remarkable progress in patients with malignant tumors (21). Preclinical trials have also shown the potential of immunotherapy in LMS (22, 23), but clinical trials indicated that immune checkpoint blockades alone are limited for patients with LMS (24, 25). Previous trials have explored the efficacy of combining immunotherapy with other agents for patients with STS (including LMS). For example, a five-year phase I/II study with an expanded phase II study of 101 patients with advanced STS has shown promising efficacy in the combination regimen with trabectedin, ipilimumab, and nivolumab (26). A subgroup analysis indicated that first-line combinatorial therapy with trabectedin, ipilimumab, and nivolumab may be more effective than standard first-line therapy, and further prospective studies are of great need to confirm this result (27). In addition, a Spanish sarcoma group phase Ib trial highlighted the efficacy of doxorubicin and dacarbazine plus nivolumab in first-line treatment of advanced LMS (28). Another phase Ib study also showed that lurbinectedin plus doxorubicin is well-tolerated with efficacy, especially in patients with LMS (29). As a result, a randomized II trial is enrolling (NCT05099666). Of note, the combination of cabozantinib and temozolomide also demonstrated meaningful clinical benefit in patients with unresectable or metastatic LMS in a phase II study (30). Furthermore, the results from a low-dose expansion cohort revealed that the addition of TTI-621 to doxorubicin showed promising clinical activity and a favorable safety profile for patients with metastatic or unresectable LMS (31).

### 3.2 The role of HIFU in the treatment of malignant tumors

As a novel modality in the treatment of t malignant tumors, HIFU was approved by the Food and Drug Administration (FDA) for treating patients with leiomyomas in 2004 (32). A recent retrospective trial showed that HIFU play a vital role in the leiomyomas for the low reintervention rate and significantly symptomatic relief (33). Previous researches also revealed the value of HIFU as a method of ablation for breast cancer (34, 35). Further study compared the histopathological changes of traditional radiofrequency ablation and HIFU, and the result showed that HIFU created smaller transition zones than traditional radiofrequency ablation, indicating a more defined area of effect with HIFU (36). In addition, several studies have demonstrated low morbidities in patients with prostate cancer who received HIFU treatment, indicating the potential use of HIFU in prostate cancer (37, 38). Of note, Yu et al. have showed that the local disease control rate was 80.6% for patients with STS after three months of HIFU treatment, which indicates that HIFU performed promising activity in patients with local unresectable recurrent STS (39). What’s more, increasing studies have explored the values of HIFU in advanced malignant tumors including pancreatic cancer, central nervous system tumors, liver cancer, ovary cancer and so on (32, 40–42).

HIFU can damage tumor cells, causing them to undergo complete coagulative necrosis and lose the ability to proliferate and metastasize. Previous studies have also demonstrated that HIFU can enhance the delivery of various therapeutic agents through multiple mechanisms, including sonoporation (transient increase in cell membrane permeability), increased vascular permeability, and triggered release from temperature-sensitive nanocarriers (32, 43, 44). Currently, most studies focus on the efficacy of HIFU as a standalone ablative treatment in malignant tumors (32, 45), while relatively few studies have explored the application of combined HIFU and systemic therapies. In our case, we reported a patient diagnosed with advanced LMS with rectal and pelvic metastasis, who experienced disease progression even after the operation and systematic therapy. The combination of HIFU, eribulin, and sintilimab was effective in controlling the disease. Of note, after the two cycles of eribulin and sintilimab, the patient’s disease remained stable, and during the third cycle of treatment, he received the combination of HIFU, eribulin, and sintilimab treatment. At this time, he achieved partial response, which suggests that HIFU may have augmented the antitumor activity of the systemic regimen. The durable response of 7.2 months, observed well beyond the immediate ablative period of HIFU, supports the hypothesis that local ablation can potentiate the effects of chemotherapy and immunotherapy. Nevertheless, larger prospective studies are required to validate this approach.

### 3.3 Implications of this case

Our patient initially achieved disease stabilization with eribulin and sintilimab alone. However, a pronounced and durable tumor reduction (PFS 7.2 months) occurred only after HIFU was added to the regimen. This temporal sequence, combined with the sustained response beyond the immediate ablation period, suggests that HIFU may have enhanced the activity of the systemic therapy, rather than merely providing a local cytoreductive effect. Notably, this prolonged disease control was achieved in a patient who had previously experienced rapid progression on anlotinib after a single cycle. As of April 2026, the patient’s OS has reached 57 months, underscoring the potential clinical value of this multimodal approach in advanced LMS. While these findings are limited to a single case and a definitive synergistic effect cannot be established, the observation aligns with the emerging evidence regarding the combination of local thermal ablation and immunochemotherapy. Future prospective studies are required to evaluate the optimal sequencing and long-term durability of response following HIFU combined with systemic agents in STS.

The precise mechanisms underlying the disease control and potential synergistic effects in this triple combination regimen of HIFU, eribulin, and sintilimab are likely multifactorial. First, HIFU exerts a direct cytoreductive effect through coagulative necrosis, rapidly reducing the local tumor burden. Beyond local ablation, thermal and mechanical stress induced by HIFU can lead to the release of damage associated molecular patterns and tumor associated antigens, potentially converting an immunologically cold tumor into a hot one. This immunomodulatory effect of HIFU provides a strong rationale for synergy with sintilimab, as the newly primed T cells can be unleashed by programmed cell death protein 1(PD-1) blockade to mount a robust systemic antitumor response. Furthermore, there is a strong biological basis for the synergy between HIFU and eribulin, as well as between eribulin and immunotherapy. HIFU induced mild hyperthermia and sonoporation at the tumor periphery can increase local vascular permeability, thereby enhancing the intratumoral delivery and concentration of eribulin. Eribulin itself possesses unique noncytotoxic properties, including the remodeling of the tumor vasculature and the reversal of epithelial to mesenchymal transition. This vascular normalization can alleviate tumor hypoxia, reduce the immunosuppressive tumor microenvironment, and facilitate the infiltration of cytotoxic T lymphocytes, thereby further synergizing with the immune checkpoint blockade provided by sintilimab. Together, this spatial and biological cooperation, where HIFU disrupts the primary tumor architecture and stimulates immunity, eribulin remodels the microenvironment and exerts cytotoxicity, and sintilimab amplifies the T cell response, likely underpins the profound and durable clinical benefit observed in our patient.

## 4 Conclusion

In summary, the integration of HIFU with eribulin and sintilimab led to a sustained response in a patient with advanced LMS who had failed multiple lines of treatment. While the 7.2-month PFS and 57-month OS are encouraging, these findings from a single-case observation should be interpreted with caution. They serve as a basis for future research to investigate the potential of local thermal ablation in enhancing systemic therapy. Further prospective clinical trials are necessary to establish the standard of care and long-term benefits of this combined strategy.

## Data Availability

The original contributions presented in the study are included in the article/supplementary material. Further inquiries can be directed to the corresponding authors.
